# Altered Functional Network Associated With Cognitive Performance in Early Parkinson Disease Measured by Eigenvector Centrality Mapping

**DOI:** 10.3389/fnagi.2020.554660

**Published:** 2020-10-16

**Authors:** Fang Cao, Xiaojun Guan, Yanqing Ma, Yuan Shao, Jianguo Zhong

**Affiliations:** ^1^Department of Radiology, Zhejiang Provincial People’s Hospital, People’s Hospital of Hangzhou Medical College, Hangzhou, China; ^2^Department of Radiology, Second Affiliated Hospital, School of Medicine, Zhejiang University, Hangzhou, China; ^3^Department of Radiology, Zhejiang Provincial People’s Hospital, People’s Hospital of Hangzhou Medical College, Hangzhou, China

**Keywords:** Parkinson disease, fMRI, eigenvector centrality, cognition, memory, executive function

## Abstract

**Objective**: To investigate relationships between whole-brain functional changes and the performance of multiple cognitive functions in early Parkinson’s disease (PD).

**Methods**: In the current study, we evaluated resting-state functional MRI (rsfMRI) data and neuropsychological assessments for various cognitive functions in a cohort with 84 early PD patients from the Parkinson’s Progression Markers Initiative (PPMI). Eigenvector centrality (EC) mapping based on rsfMRI was used to identify the functional connectivity of brain areas correlated with different neuropsychological scores at a whole-brain level.

**Results**: Our study demonstrated that in the early PD patients, scores of Letter Number Sequencing (LNS) were positively correlated with EC in the left inferior occipital gyrus (IOG) and lingual gyrus. The immediate recall scores of Hopkins Verbal Learning Test-Revised (HVLT-R) were positively correlated with EC in the left superior frontal gyrus. No correlation was found between the EC and other cognitive performance scores.

**Conclusions**: Functional alternations in the left occipital lobe (inferior occipital and lingual gyrus) and left superior frontal gyrus may account for the performance of working memory and immediate recall memory, respectively in early PD. These results may broaden the understanding of the potential mechanism of cognitive impairments in early PD.

## Introduction

Cognitive impairment is a serious and common nonmotor symptom of Parkinson’s disease (PD), which has received increasing attention in recent years. It can occur at different stages of PD, even at the time of PD diagnosis (Williams-Gray et al., 2007). In patients newly diagnosed with PD, the frequency of mild cognitive impairment (MCI) was approximately 20–40% (Yarnall et al., 2014; Aarsland et al., 2017). Early MCI is an important risk factor for dementia. The manifestations of cognitive decline in PD can be highly heterogeneous. Dysfunction in various cognitive domains has been reported in the early stages of PD before overt dementia, such as executive functions, memory, attention, visuospatial performance, and language (Yarnall et al., 2014; Aarsland et al., 2017). A previous study suggested that deficits in specific cognitive functions imposed different degrees of risk for developing dementia (Pagonabarraga and Kulisevsky, 2012). However, the potential brain mechanisms of dysfunction in the processes of diverse cognitive functions remain to be fully studied.

Several MRI studies have explored brain structural and functional network changes related to cognitive performance in PD patients. Widespread gray matter atrophy or white matter damage was observed in PD patients with relatively long disease duration (Melzer et al., 2012; Agosta et al., 2014). A few studies based on structural MRI further suggested that differences in regional brain volume may contribute to between-patient cognitive heterogeneity in PD (Gerrits et al., 2016; Gao et al., 2017). However, in the early stages of PD, structural changes were always limited and subtle. Functional MRI (fMRI) studies indicated that functional alternations in widely distributed brain networks, such as the frontoparietal network, cortico-striatal circuits, default mode network (DMN), were associated with overall cognitive decline in patients with PD (Monchi et al., 2007; Tessitore et al., 2012; Díez-Cirarda et al., 2018; Hou et al., 2018). Most of the previous fMRI studies investigated functional changes in *a priori* seed regions or subnetworks. Moreover, to our knowledge, the neuroanatomic basis of dysfunction in some specific cognitive processes was shown to be different between PD and other neurodegenerative diseases (Lee et al., 2010). The key brain nodes or networks related to deficits in various cognitive functions in early PD remained to be illuminated. Therefore, a broader and more integrated brain network beyond the known focal regions or subnetworks needs to be investigated.

As a data-driven approach, graph theory-based methods have been increasingly proposed for the analysis of brain MRI data and became a powerful tool for exploring alterations in brain functional and structural networks. Brain networks are described as graphs composed of nodes (anatomic brain regions) linked by edges (i.e., functional connections). To our knowledge, only a few studies used graph theory-based methods to evaluate cognitive impairment in PD patients (Baggio et al., 2014). In the present study, we focus on a special type of graph theory-based method that identifies nodes playing central roles within the brain network. Such nodes are characterized by a measure called “centrality”. Eigenvector centrality (EC) measures the sum of centralities of a node’s direct neighbors by assessing both the number and the quality of connections, making it sensitive to different layers in the network hierarchy (Lohmann et al., 2010; Wink et al., 2012). EC mapping (ECM) attributes a value of each voxel in the whole brain, therefore, it can objectively detect all the important brain regions that have greater connections with other parts of the brain. Unlike seed-based analysis or Independent Component Analysis (ICA), EC considers the brain as one large network rather than several subnetworks, so it is independent of *a priori* knowledge and free of researcher selection bias. ECM has proven valuable in investigating various nervous system diseases, such as Alzheimer’s disease (AD), major depressive disorder, and nicotine dependence (Binnewijzend et al., 2014; Song et al., 2016; Shen et al., 2017).

In the present study, we aimed to investigate the alterations of functional connections that were associated with the performance of multiple cognitive functions over the entire brain in early PD patients by using EC as a mapping tool.

## Materials and Methods

### Subjects

Data used in this article were obtained from the Parkinson’s Progression Markers Initiative (PPMI) database^1^. PPMI is an observational, multi-center study that has been collecting a large variety of clinical, imaging data and biologic samples to identify more biomarkers of PD progression. This research protocol was approved by local Institutional Review Boards of all the participating institutions, and written informed consent was obtained from all participants. Major inclusion criteria of early PD subjects were as follows: (1) at least two of the following symptoms: resting tremor, bradykinesia, rigidity or either asymmetric resting tremor or asymmetric bradykinesia; (2) Hoehn and Yahr stage I or II; (3) age 30 years or older; and (4) dopamine transporter deficit suggested by dopamine transporter SPECT scan. Exclusion criteria mainly included atypical PD syndromes and any medical or psychiatric condition or lab abnormality, which might preclude participation.

### Neuropsychological Assessments

Participants underwent a series of cognitive tests evaluating various cognitive functions, including: (1) Hopkins Verbal Learning Test-Revised (HVLT-R) as a measure of verbal memory; (2) Benton Judgment of Line Orientation (BJLO) test for assessing visuospatial function; (3) Semantic Fluency test mainly for reflecting executive function; (4) WMS-III Letter Number Sequencing (LNS) test as a measure of working memory; and (5) Symbol Digit Modalities Test (SDMT) for assessing attention function.

### Imaging Data Acquisition

Both structural and rest-state fMRI (rsfMRI) data were downloaded from the PPMI database. A standard MR scan was performed using a Siemens 3T TIM Trio scanner. The structural images were obtained using a 3D MPRAGE with GRAPPA T1-weighted sequence: sagittal orientation; repetition time (TR) = 2,300 ms; echo time (TE) = 2.98 ms; inversion time (TI) = 900 ms; flip angle (FA) = 9°; Field of View_(FOV)_ = 240 × 256 mm^2^; voxel size = 1 × 1 × 1 mm^3^. The rsfMRI images were acquired using an echo-planar imaging sequence with following parameters: 185 EPI volumes, with 40 ascending slices each; TR = 2,400 ms; TE = 25 ms; flip angle = 80°; slice thickness = 3.3 mm; FOV = 222 × 222 mm^2^; voxel size = 3.3 × 3.3 × 3.3 mm^3^.

### Imaging Preprocessing

Image data were preprocessed with the Data Processing and Analysis for Brain Imaging^2^, based on the Statistical Parameter Mapping software (SPM 8^3^). The first 10 volumes were discarded from the analysis. The remaining rsfMRI images were used for the following procedures: slice timing correction (using middle slice as a reference) and realignment to correct head motion, nuisance covariates regression (Friston 24 parameters, white matter, and cerebrospinal fluid signal), normalization to the standard space and resampling into a 3 × 3 × 3 mm^3^ voxel size, spatially smoothing with a Gaussian kernel of 6 mm full width at half maximum (FWHM), temporal band-pass filtering (0.01–0.1 Hz), detrending and scrubbing. Subjects were excluded if the head motion was exceeding 2 mm (displacement in the x, y, or z directions) or 2° (angular motion).

### ECM

ECM of the pre-processed rsfMRI image data was performed using fast ECM (fECM) software^4^ (Wink et al., 2012). EC measures the sum of centralities of the node’s direct neighbors, which are produced by calculating linear correlations between the fMRI time series and estimating eigenvector at each iteration. Biologically, EC can reflect the global functional importance of each voxel in the brain and has been shown to compare favorably to other centrality measures (Joyce et al., 2010). A node with a higher EC value means greater connectedness with the rest part of the brain. Compared to traditional ECM calculation methods, the time and memory required for the fECM algorithm are very small, because it computes the matrix-vector products directly from the data, without explicitly storing the connectivity matrix.

### Statistical Analysis

Demographic continuous data and neuropsychological scores were expressed as means ± standard deviations. For imaging data, the correlations between eigenvector centralities of different brain regions and neuropsychological scores were identified by linear correlation analysis performed in SPM8, age and sex as covariates. For the multiple comparison correction, Gaussian random field (voxel-level *P* < 0.001, cluster level *P* < 0.05) was employed. To further control the type I error, the Bonferroni method was further conducted in the multi-cognitive scale level.

## Results

One-hundred and one early stage PD patients had rsfMRI data. Seventeen patients were excluded due to imaging quality (including predominant head motion or scans that had not covered the whole brain). Finally, 84 subjects were included for further analysis. In the included patients, two participants were lack of SDMT scores. The detailed demographic data and neuropsychological assessment scores were listed in Table 1.

**Table 1:** Demographic and neuropsychological data of Parkinson’s disease (PD) patients.

	PD patients (*n* = 84)
Female/male	28/56
Age (years)	59.89 ± 9.99
Education (years)	15.21 ± 3.01
UPDRS (motor scores)	20.87 ± 10.31
H-Y stage	1.71 ± 0.49
HVLT-R	
Immediate recall	47.94 ± 13.63
Delayed recall	49.94 ± 14.23
Retention	50.74 ± 13.34
Recognition	53.93 ± 13.91
BJLO	12.74 ± 1.83
LNS	11.01 ± 2.71
SF	53.62 ± 11.47
SDMT	44.43 ± 9.44*

Values are expressed as means ± standard deviations or numbers of subjects. UPDRS, Unified Parkinson’s Disease Rating Scale; H-Y stage: Hoehn and Yahr stage; HVLT-R, Hopkins Verbal Learning Test-Revised; BJLO, Benton Judgment of Line Orientation test; LNS, WMS-III Letter Number Sequencing test; SF, Semantic Fluency test; SDMT, Symbol Digit Modalities Test. *SDMT scores were calculated from 82 patients.

**Figure 1:**
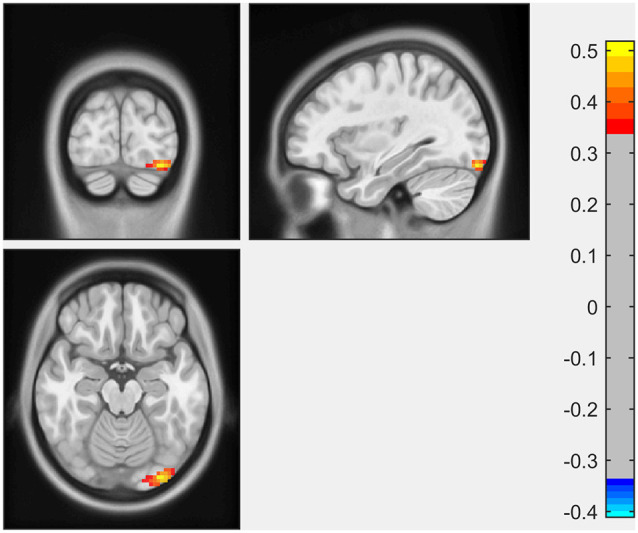
The Letter Number Sequencing (LNS) scores were positively correlated with eigenvector centrality (EC) in the left inferior occipital gyrus (IOG) and lingual gyrus.

**Figure 2:**
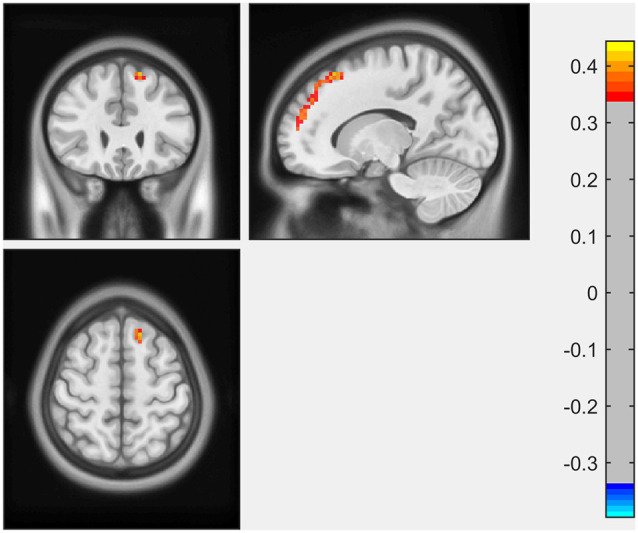
The immediate recall scores of Hopkins Verbal Learning Test-Revised (HVLT-R) were positively correlated with EC in the left superior frontal gyrus (SFG).

ECM analysis showed that EC values in the left inferior occipital gyrus (IOG) and lingual gyrus were positively correlated with the scores of the LNS test (Figure 1, Table 2). The immediate recall scores of HVLT-R were positively correlated with EC in the left superior frontal gyrus (SFG; Figure 2, Table 2). However, no significant correlations were detected between the scores of other cognitive assessments (delayed recall, retention, and recognition of HVLT-R, BJLO, SF, and SDMT) and the EC values.

**Table 2:** Eigenvector centrality (EC) of brain areas showing significant correlations with neuropsychological scores.

Cognitive scales	Cluster voxels	Brain regions	Peak intensity	MNI coordinate			*T*-value
				*x*	*y*	*z*	
LNS	57	Left inferior occipital gyrus	0.51833	−33	−90	−18	5.42
		Left lingual gyrus
HVLT-R (immediate recall)	104	Left superior frontal gyrus	0.41753	−15	27	57	4.11

After controlling the type I error at the multi-cognitive scale level, no significant correlation between EC values and cognitive scores was observed.

## Discussion

In the present study, we investigated the relationship between the performance of multiple cognitive tests and the eigenvector centrality of the whole brain. The major findings were as follows: (1) performance of working memory (LNS scores) in early PD was positively associated with EC in the left inferior occipital gyrus and lingual gyrus; and (2) EC in the left superior frontal gyrus was associated with immediate recall scores for memory in early PD patients. These results suggested that in the early stage of PD, subtle alternations in different cognitive aspects could be reflected by the disruptions of functional connectivity using ECM.

To our knowledge, this is the first study using the ECM method to evaluate the whole brain network connectivity correlated with cognitive functions in the early stage PD patients. There were only a few studies that used ECM to investigate the underlying functional network alternations associated with motor and other nonmotor symptoms (e.g., depression, dysphagia) in PD patients (Holiga et al., 2015; Lou et al., 2015; Mueller et al., 2018; de Schipper et al., 2018; Gao et al., 2019). In a study by de Schipper et al. (2018), decreased EC was shown in the occipital and frontal lobes in PD patients, which indicated diminished connectivity of these regions within the overall brain functional architecture in PD.

The Letter-Number Sequencing Test is a measure of verbal working memory. Working memory, which refers to the ability to flexibly maintain and manipulate information for a short period, is generally considered a core component of executive function (Matthews, 2015). Our study showed a positive correlation between the LNS scores with the EC in the left occipital lobe (inferior occipital gyrus, lingual gyrus), which might imply an important role of these brain regions in the working memory performance in the early PD. There was growing evidence suggesting the posterior parts of the brain, including the occipital lobe, as the key pathophysiologic regions in cognitive impairment in PD. A positron emission tomography (PET) study showed decreased metabolism in the occipito-parietal cortex was associated with cognitive dysfunction in PD (Bohnen et al., 2011). Several MRI studies showed structural atrophy (Pagonabarraga et al., 2013; Garcia-Diaz et al., 2014; Pereira et al., 2014; Wu et al., 2018; Xuan et al., 2019) and functional disruption (Olde Dubbelink et al., 2014; Fang et al., 2017; Kawabata et al., 2018) in the occipital lobe in PD patients, which might contribute to impairment in multiple cognitive functions in PD (Pagonabarraga et al., 2013; Garcia-Diaz et al., 2014; Pereira et al., 2014). Disrupted functional connectivity of the occipital region was also reported in other neurodegenerative diseases, such as Lewy body dementia and Alzheimer’s disease, suggesting a shared profile of functional changes in the cognitive disorders (Galvin et al., 2011). Conventionally, the cortical substrate for working memory/executive function tasks was predominantly attributed to the frontal region. However, working memory/executive function consisted of complex cognitive processes, and depended on distributed neural systems containing multiple brain regions (Petrides, 2015). Therefore, the term working memory is not necessarily equated with frontal function. In a task fMRI study, Ekman et al. (2014) found a significant activity decrease in the fusiform gyrus (a region adjacent to the lingual gyrus) during a verbal working-memory task between baseline and follow-up in PD with MCI, indicating that the fusiform gyrus could be responsible for the longitudinal changes of working-memory maintenance in PD patients. Similar to our results, Lee et al. (2013) found that the performance of executive function task significantly correlated with occipital gray matter volume in early non-demented PD patients, rather than with the expected frontal region. The mechanisms of relationship between changes in the occipital lobe and cognitive decline in PD still need further investigation. Molecular imaging studies suggested that the degeneration of basal forebrain cholinergic fibers projecting to the posterior parts seemed to be an important cause for PD cognitive progression (Roy et al., 2016).

Another finding in our study was the positive correlation between EC of left SFG and immediate recall scores for memory. Previous studies showed the structural and functional changes of the frontal lobe in the early stages of PD, especially SFG and middle frontal gyrus (MFG). In a recent meta-analysis of structural studies in PD, left SFG was among the brain regions with the most significant regional gray matter volume reductions (Xu et al., 2020). Generally, the temporal lobe is considered critical to the memory process, particularly episodic memory formation. However, damage in areas interconnected to the temporal lobe may also lead to memory impairment (Matthews, 2015). The frontal cortex receives inputs from all association areas including the ventral temporal lobe and limbic system (Ceccarini et al., 2019). Therefore, it also takes an important part in memory, mainly in encoding and retrieval functions. Lebedev et al. (2014) investigated rsfMRI correlate of cognitive impairment in PD patients from a large-scale network perspective. The result showed that better memory performance correlated with increased prefrontal-limbic processing. In a rsfMRI study of rigidity-dominant PD, memory scores were positively correlated with the functional connectivity between the left inferior parietal lobule (IPL) and bilateral SFG (Hou et al., 2018). The neurotransmitter basis underlying changes of frontal areas in PD was thought to be related to depleted dopaminergic circuitry. However, more and more non-dopaminergic mechanisms, such as cholinergic, serotonergic and endocannabinoid systems (Cho et al., 2017; Ceccarini et al., 2019), were reported to participate in the frontal dysfunction, which might be responsible for declines in cognitive performance in early stage of PD. Moreover, we observed a dissociation in the EC correlation with different memory subtests. A previous structural study also showed that the immediate recall scores, but not the delayed recall scores, correlated with cortical thickness in PD patients (Pereira et al., 2014). It was in keeping with the finding that memory dysfunction in early PD patients to a large degree was a deficit of immediate recall (encoding), but not of retrieval or retention (Brønnick et al., 2011).

In our present study, there was no significant correlation between the performance of other cognitive assessments (BJLO, SF, and SDMT) with EC. This might be partly due to PD patients in the current study being in an early disease stage with an only subtle decline in these cognitive functions. It is worth noting that the observed relationships between EC and working memory and immediate recall memory performance did not survive after Bonferroni adjustment in the multi-cognitive scale level. Probably, the relatively small sample size and only a few features taken into analysis in the present study might be the reason, and on other hand, the risk of high type II error might occur. As stated by historical document, the balance between type I and type II error should be paid attention to, instead of strictly controlling type I error, because type I error could be gradually eliminated when the scientific issues were widely answered and replicated by other researchers(Lieberman and Cunningham, 2009). Taken together, our explorative study, demonstrating the preliminary but important evidence for the potential coupling between brain EC alterations and neuropsychological behaviors, should help understand brain function topology during a cognitive performance in early PD patients.

Several limitations of our study should be recognized and further discussed. First, we did not include normal controls in the recent study. In the PPMI project, rsfMRI scanning was conducted at some selected sites. Therefore, only a small population of normal controls had rsfMRI images in the PPMI study (*n* = 22). Second, the neurological scales used in our study were not comprehensive enough. For example, we did not assess the language function because of the lack of specific language tests in the PPMI. Meanwhile, there are various aspects of executive functioning and memory, but our cognitive batteries only involved some specific components. Furthermore, we did not conduct a longitudinal study of fMRI data and cognitive performance. Future longitudinal studies with a larger population and combining multiple imaging modalities should be conducted to confirm our findings.

## Conclusions

In summary, our study indicates that early brain functional alternations may account for the performance of different cognitive functions in early PD patients by using ECM analysis. The left occipital lobe (inferior occipital and lingual gyrus) is related to the performance of working memory. While the left superior frontal gyrus may contribute to a deficit in immediate recall of memory.

## Data Availability Statement

All datasets presented in this study are included in the article.

## Ethics Statement

The studies involving human participants were reviewed and approved by ethics committee on human experimentation of each participating PPMI site (https://www.ppmi-info.org/about-ppmi/ppmi-clinical-sites/). Data used in the preparation of this article were obtained from the Parkinson’s Progression Markers Initiative (PPMI) database (www.ppmi-info.org/data). The patients/participants provided their written informed consent to participate in this study.

## Author Contributions

FC and JZ designed the study and wrote the first draft of the article. XG analyzed the MRI data. YM and YS assisted with interpretation of findings. All authors contributed to the article and approved the submitted version.

## Conflict of Interest

The authors declare that the research was conducted in the absence of any commercial or financial relationships that could be construed as a potential conflict of interest.

## Footnotes

^1^www.ppmi-info.org/data

^2^http://rfmri.org/dpabi

^3^http://www.fil.ion.ucl.ac.uk/spm

^4^https://github.com/amwink/bias/tree/master/matlab/fastECM
